# Implementation and effectiveness of evriMED with short messages service (SMS) reminders and tailored feedback compared to standard care on adherence to treatment among tuberculosis patients in Kilimanjaro, Tanzania: proposal for a cluster randomized controlled trial

**DOI:** 10.1186/s13063-019-3483-4

**Published:** 2019-07-12

**Authors:** Marion Sumari-de Boer, Francis M. Pima, Kennedy M. Ngowi, Geoffrey M. Chelangwa, Benson A. Mtesha, Linda M. Minja, Hadija H. Semvua, Stella Mpagama, Blandina T. Mmbaga, Pythia T. Nieuwkerk, Rob E. Aarnoutse

**Affiliations:** 1 Kilimanjaro Clinical Research Institute (KCRI), PO Box 2236, Moshi, Tanzania; 2 Department of International Health, RadboudUMC, Nijmegen, the Netherlands; 3 Department of Medical Psychology, Amsterdam UMC, Amsterdam, the Netherlands; 4 National Tuberculosis and Leprosy Programme, Dar es Salaam, Tanzania; 5 Medical psychologypsychology, AMC, AZ, 9 1105, Meibergdreef, Amsterdam, Netherlands; 6 Kibong’oto Infectious Diseases Hospital, Sanya Juu, Tanzania; 7 Department of Clinical Pharmacology, RadboudUMC, Nijmegen, the Netherlands

**Keywords:** Tuberculosis, Adherence, mHealth, Real-time medication monitoring, evriMED, Cluster randomized trial, Resource-limited setting

## Abstract

**Background:**

Adherence to tuberculosis (TB) treatment is challenging because of many factors. The World Health Organization has recommended the use of digital adherence monitoring technologies in its End TB Strategy. However, evidence on improving adherence is limited. EvriMED is a real-time medication-monitoring device which was found to be feasible and acceptable in a few studies in Asia. In Tanzania, however, there may be challenges in implementing evriMED due to stigmatization, network and power access, accuracy, and cost effectiveness, which may have implications for treatment outcome. We propose a pragmatic cluster randomized trial to investigate the effectiveness of evriMED with reminder cues and tailored feedback on adherence to TB treatment in Kilimanjaro, Tanzania.

**Methods/design:**

We will create clusters in Kilimanjaro based on level of health care facility. Clusters will be randomized in an intervention arm, where evriMED will be implemented, or a control arm, where standard practice directly observed treatment will be followed. TB patients in intervention clusters will take their medication from the evriMED pillbox and receive tailored feedback. We will use the ‘Stages of Change’ model, which assumes that a person has to go through the stages of pre-contemplation, contemplation, preparation, action, and evaluation to change behavior for tailored feedback on adherence reports from the device.

**Discussion:**

If the intervention shows a significant effect on adherence and the devices are accepted, accurate, and sustainable, the intervention can be scaled up within the National Tuberculosis Programmes.

**Trial registration:**

Pan African Clinical Trials Registry, PACTR201811755733759. Registered on 8 November 2018.

**Electronic supplementary material:**

The online version of this article (10.1186/s13063-019-3483-4) contains supplementary material, which is available to authorized users.

## Background

Tuberculosis (TB) is among the main communicable diseases causing mortality worldwide, with 10.4 million cases in 2017, of whom 1.7 million died [1]. The World Health Organization (WHO) has set targets in its 2017 ‘END TB Strategy’ for reducing TB deaths by 95% and new cases by 90% by 2035 [2]. However, the END TB Strategy is hampered by the rapidly rising rates of multi-drug-resistant (MDR) TB, with 558,000 cases in 2017 worldwide [1]. Currently, TB treatment is the cornerstone of TB control, but inadequate adherence to treatment is a major factor that leads to treatment failures or development of MDR TB. TB patients have to take four types of pills for 2 months and then two types of pills for another four months; if patients adhere to this regime, they will feel much better within the first two weeks. The number of pills and the six-month duration of therapy causes challenges to TB patients with regard to adherence. Several studies have shown that adherence among TB patients in Sub-Saharan Africa is not reaching adequate levels [3–6]. Specifically, two studies in Tanzania have shown that only 79% of TB patients reached levels of 95% adherence to TB treatment in Kilimanjaro and only 83% reached adequate adherence in Mwanza [7, 8]. Having an estimated TB incidence rate of 269 (range 127–464) per 100,000 population in 2017, Tanzania is among the countries with the highest numbers of TB cases [9]. The estimated mortality rate was 47 (range 21–83) per 100,000 population in 2017 and the proportion of TB cases with MDR TB was 0.9% [9]. Treatment success is not satisfying with rates of 90% in Tanzania in 2017 [9]. The WHO has recommended directly observed treatment (DOT) for many years to improve adherence. However, the implementation of DOT in Sub-Saharan African countries, including Tanzania, is limited, leading to adherence to treatment still being a major challenge [10]. As such, the END TB Strategy recommends to use digital adherence technologies, but with the remark that evidence that such technologies improve adherence is still limited [2].

**Fig. 1:**
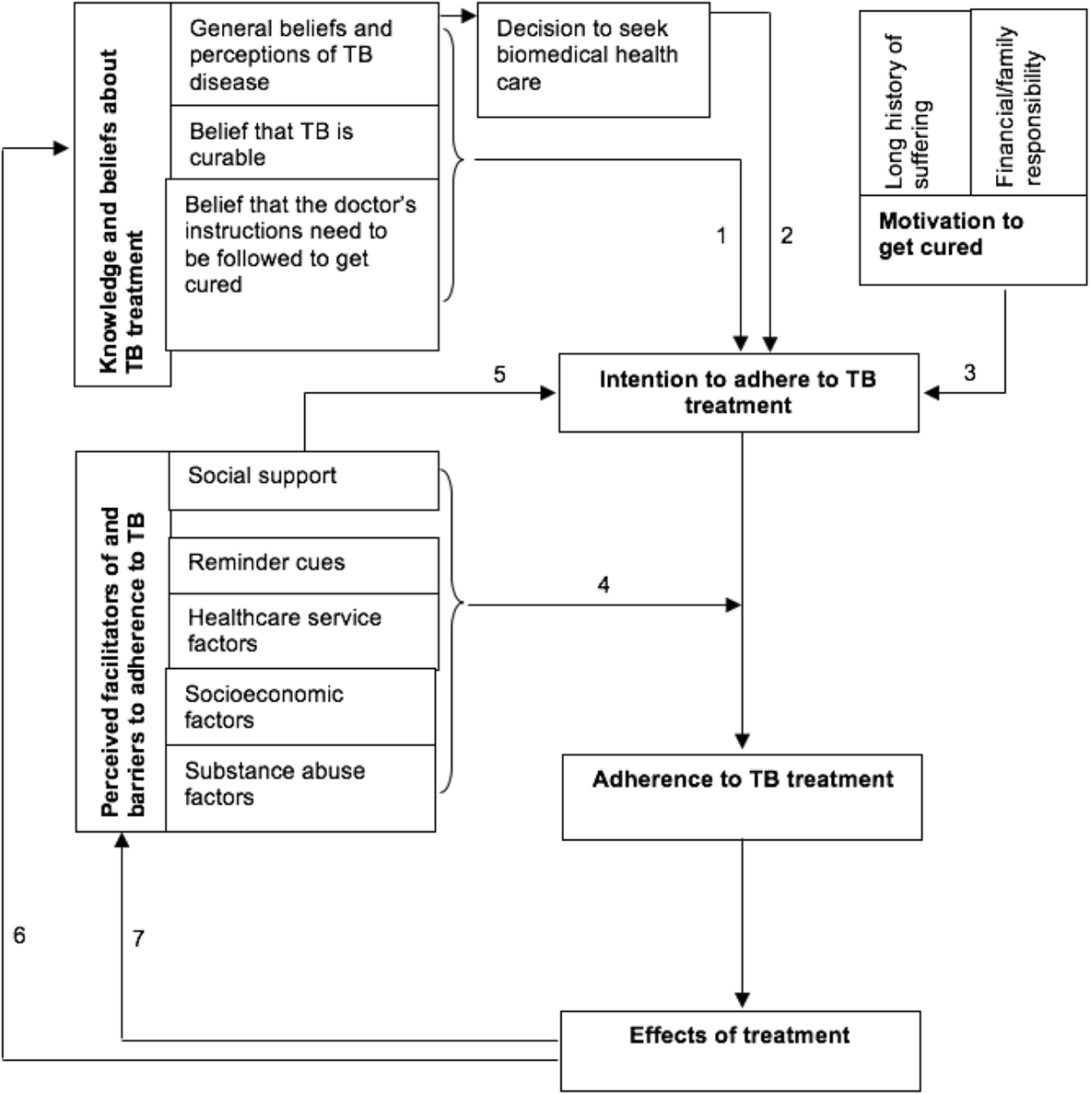
Theoretical model of treatment adherence behavior by TB patients [11]

Several factors have been found to contribute to low levels of adherence which can be summarized into patient characteristics, treatment characteristics, the patient–doctor relationship, and the organization of healthcare services [6, 11–16]. A qualitative study among TB patients in the Kilimanjaro region [11] was one of the first studies in which the relationship between adherence-influencing factors was translated into a (preliminary) model. In this model (Fig. 1), the patient’s intention to adhere to treatment is considered the most important determinant. Adherence-enabling factors include the use of reminder cues such as alarms of mobile phones [11]. According to the model, *health education interventions* are a prerequisite for adequate adherence to treatment. Health education could improve patients’ understanding of the importance of completing treatment and can be delivered through strategies involving mobile devices (Fig. 1, arrow 1) [4, 17, 18]. Reminder cues could facilitate adherence (Fig. 1, arrow 4), particularly when offered in combination with other interventions [19, 20]. According to this model, however, reminder cues alone are unlikely to make a difference in individuals who lack the intention to adhere. Also, conceding to patients’ needs and requests with respect to health care services (i.e., enhancing the ‘adherence-enabling environment’) will help patients to adhere [21–23].

Tailored feedback can target other factors that may influence adherence as determined in the model. The Stages of Change model was previously shown to be a useful model for changing health behavior, including treatment adherence [24–26]. It may provide guidance on the content of tailored feedback to enhance adherence behavior. This model proposes that health behavior change evolves through several stages: pre-contemplation, contemplation, preparation, action, maintenance, and termination. If during feedback sessions it is revealed that a patient’s level of treatment adherence has improved, this may further strengthen the participant’s adherence behavior (positive reinforcement). This may also foster higher self-efficacy (a person’s expectation that he can successfully perform a certain behavior) and internal locus of control (they believe that their own actions determine what happens to a person and not a change of external circumstances). Tailored feedback can be established through extensive counseling using *tailored feedback on individual adherence data.* Of note, such data can be captured through mHealth strategies.

mHealth intervention studies make use of mobile phones for communication with patients. Several innovative mHealth interventions have proven feasible in controlled settings; nevertheless, the evidence on feasibility and effect on adherence in real world settings in Africa is limited [27–30]. Short message service (SMS) seems to be a very promising way to assist TB patients to adhere to treatment as the penetration of simple mobile phones that use SMS for communication is high at over 80% in Tanzania [31]. Previous studies in a variety of medical conditions have shown the feasibility and acceptability of using SMS for adherence reminders in Lesotho and China [32, 33]. Effectiveness has been questioned if SMS is implemented on a large scale and for longer times. Some studies using SMS only for reminding patients have shown limited to no effect on adherence in Cameroon, China, Pakistan, and Uganda [34–37], while others have shown improvements in adherence in Malaysia and China [38, 39]. Most studies mention that SMS might cause privacy concerns, although patients have reported their appreciation of the SMS and feel like someone is taking care of them [32]. Despite varying results, authors have recommended larger studies with interactive communication between patients and health care workers and that studies should be conducted in different settings [29, 33–35, 37–39], especially in low income settings [28].

Real-time medication monitoring (RTMM) devices are a promising innovation that can be used to trigger SMS reminder cues in real time [40, 41]. RTMM works in such a way that an SMS reminder is sent to a patient only if medication is not taken at an agreed time point. Furthermore, it can create adherence reports that can be used to discuss adherence patterns with patients. In one study in Sub-Saharan Africa, RTMM (Wisepill) was shown to be feasible and it was sensitive in detecting non-adherence among MDR TB patients [42]. In Tanzania, Wisepill was considered helpful for improving adherence and was also mentioned to be a good means of storage of medications for TB patients and people living with HIV (PLHIV) [43]. Furthermore, a study in China has shown the feasibility and acceptability of a new RTMM device specifically developed for TB patients called evriMED [44]. EvriMED is different from Wisepill as it is bigger, can contain full blisters of medication, and has a different design for sending data about medication intakes. To our knowledge, no robust clinical trials have studied the efficacy and effectiveness of RTMM in improving adherence among TB patients in Sub-Saharan settings, although several studies have recommended such studies [42, 43, 45–47].

In the resource-limited setting of Kilimanjaro, mHealth may be challenging due to electrical power shortages and low availability of mobile networks. WHO, in its END TB Strategy, has advised the use of digital adherence technologies to improved adherence and specifically evriMED [2]. However, there are concerns about the implementation of evriMED in Tanzania. We believe that the appearance of the device, which is white-colored, has beeping alarm sounds and lights, and is the size of a lunchbox [48], may lead to stigmatization, leading to limited acceptance by TB patients. Furthermore, acceptance by health care providers may be hampered because they will have to use adherence reports for feedback. Also, accuracy is in question as opening of the box does not necessarily mean pill intake. Lastly, RTMM use in real-world settings in Sub-Saharan Africa has not been investigated yet. We therefore propose a pragmatic cluster randomized trial in Kilimanjaro to investigate the effectiveness of EvriMED, which will be used to generate reminder cues and adherence performance reports for tailored feedback.

## Methods/design

### Study area

Kilimanjaro Region has an area of 13,250 km^2^. It has seven districts and the capital is Moshi. In the latest census of 2012, the region had a population of about 1.6 million with a sex ratio of 94 males per 100 females. Seventy-six percent live in rural areas and 38% are aged under 15 years. The number of new TB diagnoses in Kilimanjaro in 2017 was 2681; 1126 (42%) were female. The number of previously treated patients was 54 (2%), with 21 (41%) being female. Of all cases, 263 (10%) were aged under 15 years. In Tanzania, TB patients can choose to use home-based DOT, where they assign someone in their own environment as treatment supporter, or facility-based DOT, where the patient comes to the clinic daily for medication intake [10]. Home-based DOT was chosen by 2574 (96%) patients, while 107 chose facility-based DOT [49]. There was one confirmed MDR case. We have no data on undetected cases. These numbers are based on reported cases to the National Tuberculosis and Leprosy Programme (NTLP) of Tanzania, while national WHO estimates of the number of cases is nearly double that of NTLP [50]. According to NTLP, Kilimanjaro had a treatment success rate of around 92% in 2017 with no difference between men and women [49].

### Design

This will be a pragmatic cluster randomized clinical trial with two arms. We will create 12 clusters in Kilimanjaro based on level of health care facility, numbers of reported TB cases in 2017, and location based on districts. Clusters will be randomized in an intervention arm, where evriMED will be implemented, or a control arm, where standard practice DOT will be followed. TB patients in intervention clusters will take their medication from the evriMED pillbox and receive tailored feedback on adherence during consultation. We will use the Stages of Change model to give this feedback.

### Ethics approval

This trial has been approved by the College Research and Ethics Review Committee (CRERC) of Kilimanjaro Christian Medical University College (KCMUCo) and the National Health Research Ethics Sub-Committee (NatHREC) of the National Medical Research Institute (NIMR) of Tanzania. Furthermore, the regional representatives of the Ministry of Health, the regional medical officer, and of the National Tuberculosis and Leprosy Programme, the Regional Tuberculosis Officer, have given permission to conduct the study.

### Participants

The target population consists of all diagnosed drug-susceptible adult TB patients in Kilimanjaro Region, Tanzania. Inclusion criteria are: diagnosed drug-susceptible TB patients (including smear negative patients); attending care at any of the TB treatment centers in Kilimanjaro Region; aged between 18 and 65 years; living in Kilimanjaro Region; willing to use the evriMED-device; willing to come to the clinic according to standard care; able to read and understand SMS; and able to understand and willing to sign the informed consent document. The following patients are excluded from this study: participating in other trials or previously participated in studies with electronic monitoring devices.

### Intervention

The RTMM device (evriMED1000) is a pillbox containing a SIM card. The evriMED1000 pillbox records and stores medication events (with date and time information) every time the pillbox is opened. The evriMED1000 also sends a heartbeat event every day. The heartbeat event contains device identification and shows the health of the device with technical information on battery status and signal strength. Every day, for the first opening on that day, these data are sent to a central server through the mobile network in near real-time (within 2 min). The first event and all unsent events (heartbeat and medication events) will be sent to the server at this time. If the evriMED1000 is not opened on any day, any unsent (heartbeat and medication) events will be sent at the time of the next heartbeat event.

We will configure an intake period on the server. This is the timeframe in which the medication should be taken. At the start of the intake period, an SMS message will be sent to the mobile number of the participant to remind the participant to take their medication (currently part of standard care). If the medication is not taken within one hour of the intake period, the participant will receive another reminder SMS message. The pillbox can hold enough pills as it has been designed to hold a month’s supply of up to five to ten different prescribed pills.

The intervention comprises two components. One component is the real time intervention consisting of a reminder cue through SMS in case of no intake and the other is the tailored feedback on adherence data. The reminder cues are generated as described above.

#### Attending the nurse; feedback on adherence data

Details of follow-up of patients during clinic visits are displayed in the flowchart (Table 1). After being diagnosed with TB, patients start with a consultation by the physician, which is standard care. Afterwards in the intensive and continuation phase, the participants receive usual care from the DOT nurse. The usual care by DOT nurses will be standardized in order to prevent confounding differences between control and intervention arms. Following standard care, patients in the intervention arm will receive feedback on adherence reports on each visit, which is every 2 weeks for patients in the intensive phase and after every month for patients in the continuation phase for 6 months (see Table 1). We propose a modified version of the Stages of Change model for the feedback. It combines several psychological elements, most notably corrective feedback, but education, goal setting, reinforcement, and improvement of self-efficacy will be implicated as well.

**Table 1:** Schedule of enrolment, interventions, and assessments

Visit	Visit 1	Visit 2	Visit 3	Visit 4	Visit 5	Visit 6	Visit 8	Visit 9	Visit 10
Time schedule (intensive and continuation phases)	Day 0	Day 14	Day 28	Day 42	Day 56	Day 84	Day 112	Day 140	Day 168
Provide patient information	C, R								
Oral explanation	C, R								
In- and exclusion form	C, R								
Sign informed consent	C, R								
Enrolment interview	C, R								
Questions session (including self-reported adherence)	C, R	C, R	C, R	C, R	C, R	C, R	C, R	C, R	C, R
Standardized consultation	C, R	C, R	C, R	C, R	C, R	C, R	C, R	C, R	C, R
Explanation on evriMED device	R	R	R	R	R	R	R	R	R
Provide medication	C, R	C, R	C, R	C, R	C, R	C, R	C, R	C, R	C, R
Place medication in evriMED	R	R	R	R	R	R	R	R	R
Pharmacy refill counts		C, R	C, R	C, R	C, R	C, R	C, R	C, R	C, R
Feedback sessions		R	R	R	R	R	R	R	
Evaluation									
Isoniazid urine test		C, R	C, R	C, R	C, R	C, R	C, R	C, R	C, R
Unannounced pill counts		C, R	C, R	C, R	C, R	C, R	C, R	C, R	C, R
Sputum smear test	C, R				C, R			C, R	C, R

*R* RTMM arm, *C* control arm

In the first part of the feedback session, awareness is generated by providing tailored feedback on adherence performance. A graph with medication intake-based data from the pillbox will be shown to participants so they understand where adherence could be improved. In addition, nurses will give basic education on the importance of adherence. After this phase in the session, participants will contemplate on changing their drug taking behavior. Possible pros and cons of changing behavior and barriers to improve adherence will be discussed. Nurses may offer possible solutions, such as “cues” in daily life that are to be coupled with medication intake. The third phase of a feedback session consists of the participant making decisions with respect to their behavior. An adherence goal for the next session will be set by the nurse and participant together (goal setting). The first feedback session (visit 1) will be more extensive than the subsequent feedback sessions depending on whether the patient is in the intensive phase or continuation phase.

### Measurements

#### Main objective

To answer the main objective on effectiveness of evriMED on treatment adherence and outcome, we will measure adherence to treatment in several ways and treatment outcome through sputum smear conversion and relief of symptoms. Adherence will be measured through pharmacy refill counts. This means that, at each visit, we will count the number of leftover medication that the participant brought. As such, they will be instructed to bring their leftover medication always. Furthermore, we will use self-report by asking how many pills were missed since the last visit. Furthermore, we will have adherence data from the evriMED boxes for those participants who are in the treatment arm. These data will show when the pillbox was opened, which is a proxy for medication intake. Based on that, we can count the number of days the box was opened. Adherence will be calculated by ‘Number of days when pills were taken’ divided by ‘Number of days that pills had to be taken’. Sputum smear conversion will be used as treatment outcome and measured according to standard care at month 2, month 5, and month 6 of the treatment course.

#### Secondary objectives

To measure implementation challenges, we will investigate accuracy, feasibility, and acceptability, including stigmatization. Accuracy will be measured by comparing intake based on opening of pillboxes with drug intake based on isoniazid urine strips. Feasibility will be measured through practicality, actual fit, utility, and trialability (meaning if it can be tried out on a limited basis before deciding to adopt it permanently). Also, acceptability will be measured through comfort, relative advantage, and credibility. Another aspect of acceptability is also the percentage of patients that actually agree to participate. These feasibility and acceptability outcomes will be measured through information from the evriMED system (number of intakes, number of sent reminders, network failures, battery failures, etc.) and through qualitative interviewing of participants who finished their treatment course. Furthermore, we will measure stigmatization through the Van Rie 2008 Stigma scale specific for TB [51]. This scale consists of several items a participant has to answer on a four-point Likert scale ranging from ‘strongly disagree’ to ‘strongly agree’, such as ‘Some people are afraid of those with TB’ and ‘Some people who have TB feel alone’.

### Sample size calculation and randomization

We calculated that to be able to detect a difference of mean adherence from 79% to 90%, with a power of 80% and significance of 0.05, we would need a sample size of 336 (168 in each arm). As we will use cluster randomization, we used a cluster correlation factor of 0.01. We will need to include 600 patients, 50 in 12 clusters. Considering a drop-out rate of 10%, we anticipate enrolling 660 participants in total. For a period of 8 months, all patients diagnosed with susceptible TB will be asked to participate in our study.

The whole Kilimanjaro Region was divided into 12 clusters based on location in the district, level of health care facility, and number of TB cases in 2017 in each clinic. These clusters were then randomly assigned to provide either standard of care or standard of care and pillbox. A random variable was generated in a dataset containing the clusters. Then, the clusters were sorted using a random variable. The first six clusters were assigned to intervention and the last six to control. Randomization was done using Stata 15.1.

### Data management and analyses

In this study, we believe a data monitoring committee is not needed as we are not testing a medicinal product or invasive product or device. We will use an established data management team to control data management and quality. In addition, we will not conduct interim analyses to make decisions on whether to stop the trial.

Data will be collected using electronic data capturing (EDC). We will use tablets in which a simple case report form (CRF) is pre-programmed. Data will be entered in RedCap (Research Electronic Data Capture). REDCap is a secure, web-based application designed to support data capture for research studies, providing 1) an intuitive interface for validated data entry; 2) audit trails for tracking data manipulation and export procedures; 3) automated export procedures for seamless data downloads to common statistical packages; and 4) procedures for importing data from external sources [52]. RedCap will only be accessible by authorized users. The data of participants is coded and will be treated anonymously. Data validation will be conducted using the automated query feature. Statistical tests will be performed at a two-sided significance level of 0.05. Analyses will be performed with Statistical Product and Service Solutions (IBM SPSS Statistics, Chicago, USA).

#### Baseline characteristics

Baseline characteristics data will be summarized for all participants and for the two arms. The characteristics information includes patient identification number, place of living/work, contact phone number, sex, age, referral option, phone number of treatment supporter, name of health facility, DOT option (health facility DOT or home-based DOT), and HIV status, including co-medication. We shall also take sputum smear and isoniazid urine tests for measuring treatment outcomes.

#### Primary objective

To answer the primary objective, we will compare adherence measures between the different arms. We will compare the mean adherence levels (percentage of medication taken) between arms and we will also compare percentage of participants that adhered according to the cut-off levels of 99%, 95%, 90%, and 75% (for example, 10% of patients had a mean adherence level of 99% or more). For continuous adherence levels, we will use *t*-tests or nonparametric tests depending on distribution of data. For cut-off levels, we will use χ^2^ tests. To account for the cluster effect in the analysis, we will conduct mixed effects regression models.

#### Secondary objectives

To assess accuracy, we will do unannounced pill counts and isoniazid urine tests, whereby we can measure whether pills really have been taken. The results from both tests will be compared to the data from the evriMED devices.

To assess the experience, feasibility, and acceptability of users, we will use qualitative data from interviews and focus group discussions. We will use thematic content analysis, in which recurrent themes are coded and classified in categories.

To assess technical feasibility of using evriMED in Tanzania, we will analyze data on network connection, sent and received messages, battery life of the devices, and other technical issues such as breakdowns of the boxes.

## Discussion

Adherence to treatment is a challenge for many TB patients. Many factors play a role. As such, interventions are needed that can tackle the variety of factors. EvriMED is a device for real time medication monitoring and will therefore be able to remind TB patients to take medication in case they forget. Other factors can be tackled through the suggested tailored feedback based on adherence reports from the evriMED device. The reports will be used as an entry point for discussing poor adherence and causative factors during consultation with health care providers. Therefore, we believe that evriMED might improve adherence to treatment and consequently improve TB treatment outcomes. One factor that we think might play a big role in implementing evriMED is perception of stigmatization as a consequence of using the device. Therefore, we will investigate stigmatization as a separate factor. If evriMED proves to be effective, it could be implemented on a wider scale and as such contribute to WHO’s END TB Strategy.

Stigmatization might limit the acceptability of evriMED. Several studies have shown that levels of stigmatization among TB patients are high [53–55]. We believe that the appearance of the evriMED box might contribute to higher levels of stigmatization. The box is white, is the size of a lunchbox, and also contains lights and sounds for alarming the patient to take medication. Furthermore, patients will receive reminder SMS messages. Other people seeing the box or the SMS may lead to unwanted disclosure of the condition to others. Additionally, TB patients might experience the fear of unwanted disclosure and consequent stigmatization. Our experience among PLHIV is that this fear is present based on previous pilot studies where we used SMS and RTMM [43]. Some participants of those studies mentioned actual situations of unwanted disclosure of their HIV status and the fear of stigmatization. To our knowledge, previous studies on using evriMED among TB patients in Sub-Saharan Africa have not been performed while WHO and other studies highly advocate for using RTMM and specifically evriMED. As we will measure stigmatization as a separate factor in our trial, we will be able to show whether stigmatization is a main challenge in implementing evriMED in our setting (Additional file 1).

### Limitations

We expect some limitations in our study. A well-known issue with digital medication monitoring is that the pillbox being opened is not synonymous with real medication intake. Once participants know how the systems works exactly, they may continuously cheat by opening the box but not taking medication. Only direct measurements of drugs in the body can confirm medication intake. Previous studies have shown, however, that medication monitors are valid for measuring intake [42, 56]. Also, other adherence-measuring methods like self-reporting and pharmacy refill counts are known to overestimate adherence [42]. As one of our outcome parameters is accuracy through measuring drug levels in urine with isoniazid strips, we will be able to conclude for evriMED whether they are accurate enough for monitoring medication intake.

As we will make use of SMS, only patients who are in possession of a mobile phone and can read and understand SMS can be included. This means that all patients without a mobile phone will be excluded from the study, leading to a selection of patients. As having a mobile phone might be a proxy for socio-economic-status, the representativeness of the sample is questionable. We will try to overcome this by providing a mobile phone to those who do not have one. Unfortunately, for now, we cannot overcome the selection of those who can read and understand SMS. However, if we find our intervention to be feasible, we might expand the system by using interactive voice response calling. In such a system, the patient is called and a computer reads the SMS aloud.

One risk in our study is worsening of mobile network availability. We will use international SIM cards that can roam over different networks. Users of evriMED devices and SMS may have different providers. Mobile network availability is not always stable.

For the nurses to be able to get the adherence reports, connection to the internet is needed and that might be another challenge. In cases of poor network connection, however, we may decide to purchase routers with better antennas to provide better internet to the sites. Besides limited network connection, there might also be limitations in power availability, with periods in which power availability is a problem. This may lead to challenges in charging the evriMED devices and the mobile phone. If this is a long-term problem, we will have to consider supplying participants with simple solar chargers.

### Strengths

As this is a pragmatic cluster randomized trial, we can look at both effectiveness and implementation of the device. Effectiveness has not been proven yet, but because of the WHO’s recommendation to use digital technologies to improve adherence, we believe it is important to also investigate implementation factors such as accuracy, feasibility, and acceptability. Another strength is the close collaboration with the NTLP as this may assist in further scale up of the intervention if proven effective.

### Dissemination

We anticipate that this project will conclude that evriMED will improve adherence to treatment among TB patients. We will disseminate the results to several stakeholders in the field of TB and hope that TB patients will profit from the results through changes in policies. This means that evriMED should become part of standard practice in management and treatment of TB. This can be based on patient preferences also.

In addition, other patient groups such as PLHIV and diabetic patients may profit from our results and therefore researchers in those fields should be informed to investigate the effect in those patient groups. We think that results can easily be extrapolated to TB patients in Sub-Saharan Africa, especially in Eastern Africa.

#### Community advisory board

The project will be promoted among TB patients through the existing community advisory board of TB in Moshi, Tanzania. During their scheduled meetings, we will give a presentation about the project beforehand. In addition, once a year we will give a report on the project and at the end we will present the results.

#### TB patients

At the end of the study, we will develop an information leaflet for participants of the study and other TB patients in the participating clinics describing the results of the study. Through this, TB patients will become aware of the importance of the study. In addition, we will develop posters with the same information to be used in those clinics.

#### Peer-reviewed papers

We will write and publish papers based on the results from our study. The papers will be published in peer-reviewed journals that make them available for review by students and other researchers.

#### Conference proceedings

We will write abstracts based on the findings that we will send to international and national conferences. Funding within our project is for two international conferences. In addition, we will apply for scholarships to visit more conferences to be able to present the results. In addition, we will visit national conferences to present our findings. The target population of those conferences are mainly researchers and health care staff.

#### East African Consortium of Clinical Research

KCRI is part of the East African Consortium of Clinical Research. As regular meetings will take place within the consortium, we will present our findings there. The target population are researchers within EACCR. In addition, if other existing networks will allow us, we will present our findings there also.

#### Policy briefing

We will write a policy briefing on the results of the project. This will contain information and implications for policy written in understandable language for policy makers. This policy briefing will be sent to local policy makers in Moshi, national policy makers (i.e., ministry of health, NTLP), and international policy makers, such as big foundations like the Global Fund, Bill and Melinda Gates Foundation, and PEPFAR.

### Conclusions

If evriMED, used for SMS reminders and tailored feedback during clinic visits, is shown to improve adherence to TB treatment and treatment outcomes, and is acceptable, feasible, and accurate, it could be recommended for standard card among TB patients in Sub-Saharan Africa. Further implementation studies will then be needed to explore factors that may hinder implementation of evriMED if used on a wider scale.

## Trial status

The current protocol version is 1.0 as of 5 February 2019. Recruitment has not started. We expect to start recruitment on 1st June 2019 and finish by 29 February 2020.

## Additional file

Additional file 1: SPIRIT 2013 checklist: Recommended items to address in a clinical trial protocol and related documents. (PDF 71 kb)

## Data Availability

Not applicable.

## References

[1] World Health Organization (2018). WHO global tuberculosis report executive summary 2018.

[2] World Health Organization. Guidelines for treatment of drug-susceptible tuberculosis and patient care (2017 update). April 2017. Geneva, Switzerland. Available from: https://www.who.int/tb/publications/2017/dstb_guidance_2017/en/.

[3] Kisambu J, Nuwaha F, Sekandi JN (2014). Adherence to treatment and supervision for tuberculosis in a DOTS programme among pastoralists in Uganda. Int J Tuberc Lung Dis..

[4] Kaona FAD, Tuba M, Siziya S, Sikaona L (2004). An assessment of factors contributing to treatment adherence and knowledge of TB transmission among patients on TB treatment. BMC Public Health.

[5] Kebede A, Wabe NT (2012). Medication adherence and its determinants among patients on concomitant tuberculosis and antiretroviral therapy in south west Ethiopia. N Am J Med Sci.

[6] Woimo TT, Yimer WK, Bati T, Gesesew HA (2017). The prevalence and factors associated for anti-tuberculosis treatment non-adherence among pulmonary tuberculosis patients in public health care facilities in South Ethiopia: a cross-sectional study. BMC Public Health.

[7] van den Boogaard J, Lyimo RA, Boeree MJ, Kibiki GS, Aarnoutse RE (2011). Electronic monitoring of treatment adherence and validation of alternative adherence measures in tuberculosis patients: A pilot study. Bull World Health Organ.

[8] Kidenya BR, Mshana SE, Gerwing-Adima L, Kidola J, Kasang C (2017). Drug adherence and efficacy of smear microscopy in the diagnosis of pulmonary tuberculosis after 2 months of medication in North-western Tanzania. Int J Infect Dis.

[9] World Health Organization (2018). Tuberculosis country factsheet: United Republic of Tanzania.

[10] Van Den Boogaard J, Lyimo R, Irongo CF, Boeree MJ, Schaalma H, Aarnoutse RE (2009). Community vs. facility-based directly observed treatment for tuberculosis in Tanzania’s Kilimanjaro Region. Int J Tuberc Lung Dis..

[11] Van den Boogaard J, Boeree MJ, Kibiki GS, Aarnoutse RE (2011). The complexity of the adherence-response relationship in tuberculosis treatment: Why are we still in the dark and how can we get out?. Trop Med Int Heal.

[12] Adane AA, Alene KA, Koye DN, Zeleke BM. Non-adherence to anti-tuberculosis treatment and determinant factors among patients with tuberculosis in northwest Ethiopia. PLoS One. 2013;8(11):e78791. 10.1371/journal.pone.0078791 PMC3823971 24244364

[13] Tola HH, Garmaroudi G, Shojaeizadeh D, Tol A, Yekaninejad MS, Ejeta LT, Kebede A, Kassa D (2017). The effect of psychosocial factors and patient s ’ perception of tuberculosis treatment non-adherence in Addis Ababa, Ethiopia. Ethiop J Heal Sci.

[14] Gebreweld FH, Kifle MM, Gebremicheal FE, Simel LL, Gezae MM, Ghebreyesus SS (2018). Factors influencing adherence to tuberculosis treatment in Asmara, Eritrea: a qualitative study. J Heal Popul Nutr.

[15] Méda ZC, Lin YT, Sombié I, Maré D, Morisky DE, Chen YMA (2014). Medication-adherence predictors among patients with tuberculosis or human immunodeficiency virus infection in Burkina Faso. J Microbiol Immunol Infect.

[16] Ali AOA, Prins MH (2016). Patient non adherence to tuberculosis treatment in Sudan: Socio demographic factors influencing non adherence to tuberculosis therapy in Khartoum State. Pan Afr Med J.

[17] Dick JLC (1997). Shared vision - a health education project designed to enhance adherence to anti-tuberculosis treatment. Int J Tuberc Lung Dis..

[18] Liefooghe R, Suetens C, Meulemans H, Moran MB, De Muynck A (1999). A randomised trial of the impact of counselling on treatment adherence of tuberculosis patients in Sialkot, Pakistan. Int J Tuberc Lung Dis..

[19] Nhavoto JA, Gronlun A, Klein GO. Mobile health treatment support intervention for HIV and tuberculosis in Mozambique : Perspectives of patients and healthcare workers. PLoS One. 2017;12(4):1–13. 10.1371/journal.pone.0176051 PMC5395223 28419149

[20] Bediang G, Stoll B, Elia N, Abena JL, Nolna D, Chastonay P (2014). SMS reminders to improve the tuberculosis cure rate in developing countries (TB-SMS Cameroon): A protocol of a randomised control study. Trials..

[21] Mesfin MM, Newell JN, Walley JD, Gessessew A, Tesfaye T, Lemma F (2009). Quality of tuberculosis care and its association with patient adherence to treatment in eight Ethiopian districts. Health Policy Plan.

[22] Jaiswal A, Singh V, Ogden JA, Porter JDH, Sharma PP, Sarin R (2003). Adherence to tuberculosis treatment: Lessons from the urban setting of Delhi, India. Trop Med Int Heal..

[23] Sagbakken M, Frich JC, Bjune G (2008). Barriers and enablers in the management of tuberculosis treatment in Addis Ababa, Ethiopia: A qualitative study. BMC Public Health.

[24] Prochaska JO, Velicer WF (1997). The transtheoretical model of health behavior change. Am J Health Promot.

[25] Willey C, Redding C, Stafford J, Garfield F, Geletko S, Flanigan T, Melbourne K, Mitty J, Caro JJ (2000). Stages of change for adherence with medication regimens for chronic disease: development and validation of a measure. Clin Ther.

[26] Kavookjian J, Berger BA, Grimley DM, Villaume WA, Anderson HM, Barker KN (2005). Patient decision making: Strategies for diabetes diet adherence intervention. Res Soc Adm Pharm.

[27] Liu Q, Abba K, Alejandria MM, Sinclair D, Balanag VM, Lansang MAD. Reminder systems to improve patient adherence to tuberculosis clinic appointments for diagnosis and treatment. Cochrane Database Syst Rev. 2014;2014(11):CD006594. 10.1002/14651858.CD006594.pub3 PMC4448217 25403701

[28] Marcolino MS, Oliveira JAQ, D’Agostino M, Ribeiro AL, Alkmim MBM, Novillo-Ortiz D (2018). The impact of mHealth interventions: Systematic review of systematic reviews. JMIR Mhealth Uhealth.

[29] Nglazi MD, Bekker L-G, Wood R, Hussey GD, Wiysonge CS (2013). Mobile phone text messaging for promoting adherence to anti-tuberculosis treatment: a systematic review protocol. Syst Rev.

[30] Ngwatu BK, Nsengiyumva NP, Oxlade O, Mappin-Kasirer B, Nguyen NL, Jaramillo E (2018). The impact of digital health technologies on tuberculosis treatment: a systematic review. Eur Respir J.

[31] Tanzania Communications Regulatory Authority (2017). Quarterly communications statistics report.

[32] Hirsch-Moverman Y, Daftary A, Yuengling KA, Saito S, Ntoane M, Frederix K (2017). Using mHealth for HIV/TB treatment support in Lesotho. J Acquir Immune Defic Syndr.

[33] Lei X, Liu Q, Wang H, Tang X, Li L, Wang Y (2013). Is the short messaging service feasible to improve adherence to tuberculosis care? A cross-sectional study. Trans R Soc Trop Med Hyg.

[34] Bediang G, Stoll B, Elia N, Abena J-L, Geissbuhler A (2018). SMS reminders to improve adherence and cure of tuberculosis patients in Cameroon (TB-SMS Cameroon): a randomised controlled trial. BMC Public Health.

[35] Hermans SM, Elbireer S, Tibakabikoba H, Hoefman BJ, Manabe YC (2017). Text messaging to decrease tuberculosis treatment attrition in TB-HIV coinfection in Uganda. Patient Prefer Adherence.

[36] Mohammed S, Glennerster R, Khan AJ. Impact of a daily SMS medication reminder system on tuberculosis treatment outcomes: A randomized controlled trial. PLoS One. 2016;11(11):e0162944. 10.1371/journal.pone.0162944 PMC5089745 27802283

[37] Liu X, Lewis JJ, Zhang H, Lu W, Zhang S, Zheng G (2015). Effectiveness of electronic reminders to improve medication adherence in tuberculosis patients: A cluster-randomised trial. PLoS Med.

[38] Abdulrahman SA, Rampal L, Ibrahim F, Radhakrishnan AP, Shahar HK, Othman N (2017). Mobile phone reminders and peer counseling improve adherence and treatment outcomes of patients on ART in Malaysia: A randomized clinical trial. PLoS One.

[39] Fang X-H, Guan S-Y, Tang L, Tao F-B, Zou Z, Wang J-X (2017). Effect of short message service on management of pulmonary tuberculosis patients in Anhui Province, China: A prospective, randomized, controlled study. Med Sci Monit.

[40] Subbaraman R, Mayer K, Muslimenta A, Thomas B (2018). Digital adherence technologies for the treatment of tuberculosis: landscape and research priorities. BMJ Glob Health.

[41] Nsengiyumva NP, Mappin-Kasirer B, Oxlade O, Bastos M, Trajman A, Falzon D, et al. Evaluating the potential costs and impact of digital health technologies for tuberculosis treatment support. Eur Respir J. 2018;1801363 Available from: http://erj.ersjournals.com/lookup/doi/10.1183/13993003.01363-2018. 10.1183/13993003.01363-2018 PMC6214576 30166325

[42] Bionghi N, Daftary A, Maharaj B, Msibi Z, Amico KR, Friedland G (2018). Pilot evaluation of a second-generation electronic pill box for adherence to Bedaquiline and antiretroviral therapy in drug-resistant TB/HIV co-infected patients in KwaZulu-Natal, South Africa. BMC Infect Dis.

[43] Sumari-de Boer IM, van den Boogaard J, Ngowi KM, Semvua HH, Kiwango KW, Aarnoutse RE (2016). Feasibility of real time medication monitoring among HIV Infected and TB patients in a resource-limited setting. AIDS Behav.

[44] Liu X, Blaschke T, Thomas B, De Geest S, Jiang S, Gao Y (2017). Usability of a medication event reminder monitor system (MERM) by providers and patients to improve adherence in the management of tuberculosis. Int J Environ Res Public Health.

[45] Haberer JE, Kahane J, Kigozi I, Emenyonu N, Hunt P, Martin J (2010). Real-time adherence monitoring for HIV antiretroviral therapy. AIDS Behav.

[46] DeSilva MB, Gifford AL, Xu K, Li Z, Feng C, Brooks M, et al. Feasibility and acceptability of a real-time adherence device among HIV-positive IDU patients in China. (Special Issue: Using mobile health technology to improve HIV care for persons living with HIV and substance abuse.). AIDS Res Treat. 2013;957862(16). 10.1155/2013/957862 PMC3730150 23956851

[47] Sabin LL, DeSilva MB, Gill CJ, Li Z (2015). Improving adherence to antiretroviral therapy with triggered real time text message reminders: The China through Technology Study (CATS). J Acquir Immune Defic Syndr.

[48] Stop TB Partnership (2017). TB medication adherence & treatment outcomes.

[49] National Tuberculosis and Leprosy Programme (2017). Tuberculosis data from Kilimanjaro, Tanzania.

[50] World Health Organization (2016). United Republic of Tanzania: Tuberculosis profile.

[51] Van Rie A, Sengupta S, Pungrassami P, Balthip Q, Choonuan S, Kasetjaroen Y (2008). Measuring stigma associated with tuberculosis and HIV/AIDS in southern Thailand: Exploratory and confirmatory factor analyses of two new scales. Trop Med Int Health..

[52] Harris PA, Taylor R, Thielke R, Payne J, Gonzalez N, Conde JG (2009). Research electronic data capture (REDCap)--a metadata-driven methodology and workflow process for providing translational research informatics support. J Biomed Inform.

[53] Hassard S, Ronald A, Angella K (2017). Patient attitudes towards community-based tuberculosis DOT and adherence to treatment in an urban setting; Kampala, Uganda. Pan Afr Med J..

[54] Kipp AM, Pungrassami P, Stewart PW, Chongsuvivatwong V, Strauss RP, Van Rie A (2011). Study of tuberculosis and AIDS stigma as barriers to tuberculosis treatment adherence using validated stigma scales. Int J Tuberc Lung Dis.

[55] Chowdhury MRK, Rahman MS, Mondal MNI, Sayem A, Billah B (2015). Social impact of stigma regarding tuberculosis hindering adherence to treatment: A cross sectional study involving tuberculosis patients in Rajshahi City, Bangladesh. Jpn J Infect Dis.

[56] Vrijens B, Tousset E, Rode R, Bertz R, Mayer S, Urquhart J (2005). Successful projection of the time course of drug concentration in plasma during a 1-year period from electronically compiled dosing-time data used as input to individually parameterized pharmacokinetic models. J Clin Pharmacol.

